# Effect of Annealing Temperature on the Microstructure, Texture, and Properties of Hot-Rolled Ferritic Stainless Steel with Preferential *α*-Fiber Orientation

**DOI:** 10.3390/ma19020293

**Published:** 2026-01-11

**Authors:** Rongxun Piao, Jinhui Zhang, Gang Zhao, Junhai Wang

**Affiliations:** 1 School of Mechatronics Engineering, Anhui University of Science and Technology, Huainan 232001, China; 18375372135@163.com; 2 Anhui Intelligent Mine Technology and Equipment Engineering Research Center, Huainan 232001, China; 3 Technical Research and Development Center, Shandong Taishan Steel Group Co., Ltd., Jinan 271100, China; caocunzg@163.com

**Keywords:** ferritic stainless steel, annealing, texture, mechanical properties, plastic strain ratio

## Abstract

For hot-rolled ferritic stainless steels with preferential α-fiber texture, the strong α-fiber texture is retained after annealing, greatly affecting the texture and plastic formability during the subsequent cold-rolling process. For optimizing the texture of hot-rolled steels toward the favorable γ-fiber type, it is essential to control the annealing temperature in the annealing process. To investigate the evolution of the microstructure, texture, and properties of hot-rolled ferritic stainless steel with preferential α-fiber orientation, a series of annealing tests was performed at the lab scale at 800, 840, 880, 910, 930, and 950 °C for 3 min. The microstructure, texture, and grain boundary characteristics of the tested samples were analyzed using optical microscopy (OM) and electron back-scattered diffraction (EBSD). The mechanical properties and plastic strain ratio (r-value) were determined through universal tensile testing. The results show that at temperatures above 840 °C, more than 93% of recrystallization occurs, leading to significant microstructural refinement. The α-fiber texture intensity typically diminishes with rising temperature, whereas the γ-fiber texture initially weakens during the early stages of recrystallization (below 840 °C) and subsequently exhibits a slight increase at higher temperatures. The improved formability of the material is mainly attributed to microstructural refinement and texture refinement, as reflected by the I(γ)/I(α) texture intensity ratio. At an annealing temperature of 930 °C, the I(γ)/I(α) ratio peaks at 0.85, static toughness is maximized, the strain-hardening exponent (*n*) reaches a high value of 0.28, and the maximum average plastic strain ratio ($\bar{r}$) is 0.96. This result represents the optimum balance between mechanical properties and formability, making it suitable for subsequent cold-rolling.

## 1. Introduction

Owing to high corrosion resistance, excellent mechanical properties, and low manufacturing cost, ferritic stainless steels (FSSs) are widely used for various applications such as kitchenware, household appliances, automotive exhaust systems, and critical industrial components [1,2]. However, the formability of FSSs is generally inferior to that of austenitic stainless steel and low-carbon steel, which limits their manufacturing and application in more fields [3]. The formability of FSS is commonly evaluated in terms of the plastic strain ratio (r-value), which is strongly related to texture evolution and uniform grain size distribution in the microstructure [4]. Usually, ferritic stainless steel with a γ-fiber texture (<111>//ND) is less prone to slip in the thickness direction when subjected to deformation, such as tensile deformation of sheets, while deformation is least likely to occur in the thickness direction [5], resulting in a high r-value and indicating high formability. On the other hand, for the α-fiber texture, the direction of the crystal <001> is parallel to the normal direction (ND) of the sheets. When ferritic stainless steel with α-fiber texture is subjected to tensile deformation, slip is prone to occur in the thickness direction, and deformation is most likely to occur in the thickness direction, indicating poor formability [6]. Therefore, in order to achieve better formability, ferritic stainless steel should be designed with more γ-fiber texture (<111>//ND) instead of an α-fiber texture.

In ultra-low carbon ferritic stainless steel, the final recrystallization texture is predominantly controlled by the hot-rolled or hot band texture, rather than by metallurgical conditions such as steel chemistry or the existence of precipitates [7,8]. The hot band typically exhibits a strong {001}//ND texture, which influences the texture evolution and formability throughout the entire manufacturing process [9,10]. In particular, the {001}<110> orientation, known as an unfavorable α-fiber texture, is not conducive to the formation of a strong γ-fiber texture during further processing, resulting in a low r-value [8]. The hot band annealing treatment is an effective way to diminish texture sharpness, thereby improving the subsequent cold-rolling and the final recrystallization textures significantly, which improves formability of FSS [9]. Du et al. [8] investigated the effect of hot band annealing processes on microstructure, texture, and grain boundary character of Nb-bearing ferritic stainless steel. The results show that after continuous annealing at 960 °C for 5 min, a weak γ-fiber texture was formed, and some of the α-fiber texture originating from hot-rolling was eliminated, but a relatively strong α-fiber texture remained. After cold-rolling and recrystallization annealing, a more favorable γ-fiber texture was achieved, which led to an improvement in drawability with an average r-value as high as 1.73. The statistical distribution of overlapping coincidence site lattice (CSL) boundaries revealed that the main CSL boundaries of the hot band sheet after continuous annealing were characterized by ∑3 and ∑13b grain boundaries, with fractions of 0.058 and 0.071, respectively. Bai et al. [11] explored the effect of hot band annealing temperature of 900, 950, 1000, and 1050 °C on the microstructure, texture evolution, and formability of Sn-bearing FSS hot-rolled bands after cold-rolling and annealing. The results indicate that a moderate rise in annealing temperature promotes the formation and growth of recrystallized grains with uniform equiaxed γ-fiber texture while reducing the number of grains with unfavorable α-fiber orientation {001} <110>, and the maximum r-value of 1.71 was achieved at hot band annealing temperature of 950 °C, which can be attributed to the high intensity γ-fiber texture and the formation of uniformly equiaxed recrystallized grains. Patra et al. [12] investigated the influence of hot band annealing on surface ridging and formability in Type 409L Ti-bearing cold-rolled sheets. The results show that annealing the hot band at 940 °C effectively promotes the recrystallization and refinement of coarse cube-oriented grains, and the intensity of the γ-fiber texture is thereby significantly reducing ridging and improves the sheet’s formability. At 940 °C, the 409L cold-rolled sheet exhibits an ultimate tensile strength of 435 MPa, a yield strength of 257 MPa, and an elongation of 34%.

So far, many studies have focused on analyzing the relationship between microstructure, texture evolution, and formability of cold-rolled FSS with annealing treatment, but systematic research on the annealing of hot-rolled steels is limited. Although annealing of hot-rolled steels has been confirmed to improve the formability of FSS [13], for hot-rolled steels with preferential α-fiber texture, a strong α-fiber texture is still retained after annealing [5,6], and the behavior of texture evolution for α-fiber and γ-fiber texture has not been explored yet.

In our present study, the microstructure and texture evolution, and mechanical properties of Type 409L hot-rolled stainless-steel sheets with preferential α-fiber texture under varying annealing temperatures were investigated. Also, the formability of materials was evaluated through the measurements of important indexes of plastic strain ratio and earring coefficient (∆r). This research aims to systematically explore the influence of annealing temperature on the microstructure and texture evolution behavior (including grain size distribution and characteristics of grain boundary), mechanical properties, as well as strain hardening exponent (*n*-value) and plastic strain ratio (r-value) of hot-rolled sheets during annealing, with the ultimate goal of identifying optimal conditions for subsequent cold-rolling.

## 2. Materials and Methods

An industrial product in the form of hot-rolled sheets of 409L grade ferritic stainless steel (FSS) was used in the present study. The preparation process of hot-rolled sheets is as follows: a 200 mm thick cast slab was heated at 1200 °C for 3 h, and then hot-rolled to a 3 mm thick sheet through seven passes of rough rolling and seven passes of finishing rolling in a rough mill and finishing mill while maintaining the final rolling temperature of around 870 °C. The chemical compositions (in weight percent, %) of the investigated steel were C0.011, N0.009, Si0.59, Mn0.17, Cr11.31, and Ti0.22, which were detected using SPECTROLAB S optical emission spectrometer (SPECTRO Analytical Instruments Co., Ltd., Kleve, Germany) according to GB/T 11170-2008 [14]. Then, the specimens were subjected to leveling treatment and wire-cut into a rectangular shape (100 mm × 30 mm) of specimens. In order to simulate the industrial condition [5,11], the specimens were annealed at 800, 840, 880, 910, 930, and 950 °C for 3 min, with a heating rate of 10 °C/s, and cooled by water quenching.

After annealing, the specimens were wire-cut into a specific size for the subsequent microstructure and texture analysis and mechanical property test. In microstructure and texture analysis, a 5 mm × 10 mm sample was sectioned. For the microstructure analysis, the cross section of the sample was analyzed by DMM490C optical microscope (OM, Caikang Optical Instrument Co., Ltd., Shanghai, China) after etching with a solution of 30 mL HCL + 150 mL C_2_H_5_OH +10 g FeCl_3_. For the texture analysis, the specimen for electron backscattering diffraction (EBSD) measurements was made by electrolytic polishing in a solution of 6% HClO_4_ + 34% C_4_H_9_OH + 60% CH_3_OH under 25 V. The polished surface on the RD-ND plane of the rolled and annealed specimens was scanned using an EDAX Hikari Plus instrument (AMETEK, Mahwah, NJ, USA). At least 1.4 mm × 1.0 mm areas were used for the EBSD analysis from each specimen with a step size of 2 μm. The crystallographic orientations of grains inside 409L hot-rolled specimens were analyzed on the basis of the orientation distribution functions (ODFs) at the Φ2 = 45° section of the Euler space. The ferrite grain size distribution was measured in grain regions containing at least 500 grains in each specimen.

**Figure 1:**
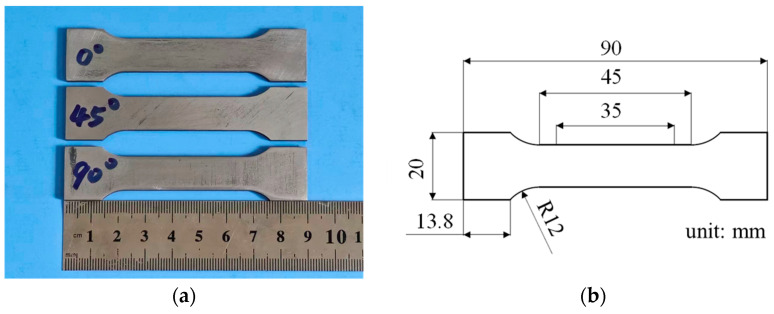
(**a**) Tensile test specimen and (**b**) its dimensional specifications.

For the mechanical properties test, based on the ASTM E8/E8M-24 (GB/T 228.1-2021 [15]) standard, tensile specimens were taken in the rolling direction (at 0°) with the shape and size as shown in Figure 1. The parallel segment was 45 mm long, and the gauge section was 35 mm in length. Tensile tests were conducted using a UTM 5105 universal testing machine (Shenzhen SUNS Technology Stock Co., Ltd., Shenzhen, China) at a speed of 1 mm/min, and the strain rate was $4\times 10^{-4}s^{-1}$. An NCM-2D noncontact video extensometer was used for the accurate measurement of strain. The tensile properties of test steels were determined by taking the average of three specimens. For the plastic strain ratio(r) and anisotropy index (∆r) measurement, according to the ASTM E517-24 (GB/T 5027-2016 [16]) standard, the tensile specimens were prepared at 0°, 45°, and 90° along the rolling direction. When the tensile strain reaches 15%, the ratio of the true plastic strain in the width direction to the true plastic strain in the thickness direction of a specimen was measured as the plastic strain ratio (r). The two specimens in each direction were tested at 15% elongation to determine their average plastic strain ratio ($\bar{r}$) and anisotropy index (∆r).

## 3. Results and Discussions

### 3.1. Microstructure

**Figure 2:**
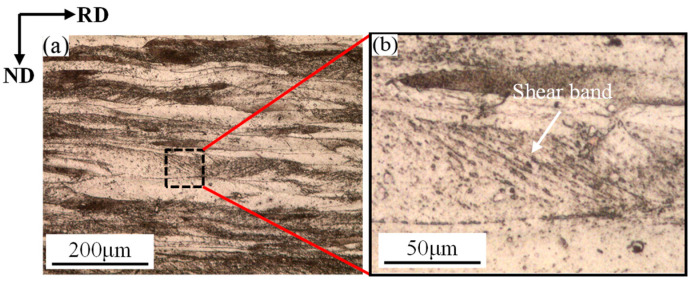
(**a**) Microstructure of 409L hot-rolled specimens; (**b**) magnified view.

Figure 2 shows the microstructure of 409L hot-rolled specimens. As shown in the figure, the microstructure of 409L exhibits a typical ribbon-like fiber features deformation structure [8], with grains elongated along the rolling (RD) direction. Meanwhile, due to the shear deformation generated by the friction between the rolling mill and the surface of the plate during the hot-rolling process, a shear band structure can be clearly observed inside the specimen, with the shear band forming at an angle of about 30° to the rolling direction.

**Figure 3:**
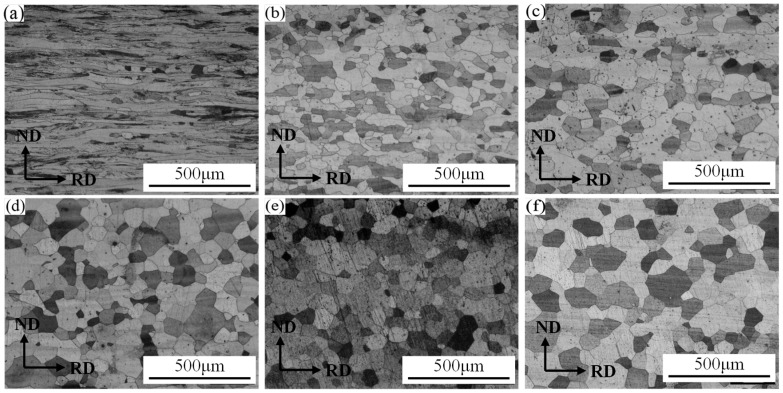
Microstructure of 409L annealed at different temperatures: (**a**) 800 °C; (**b**) 840 °C; (**c**) 880 °C; (**d**) 910 °C; (**e**) 930 °C; (**f**) 950 °C.

**Figure 4:**
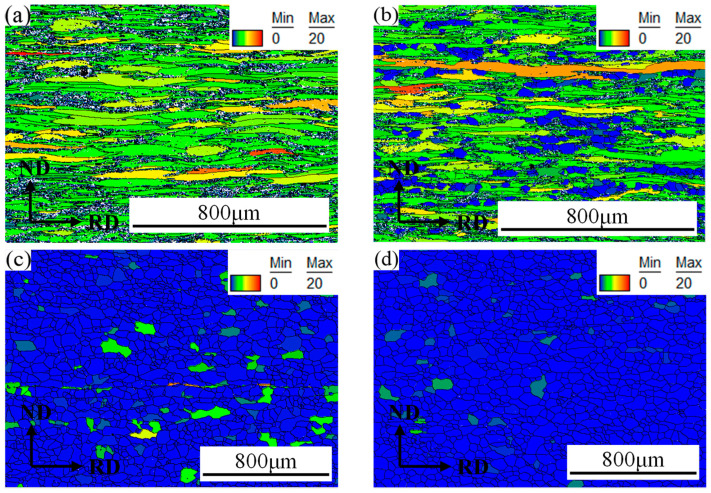

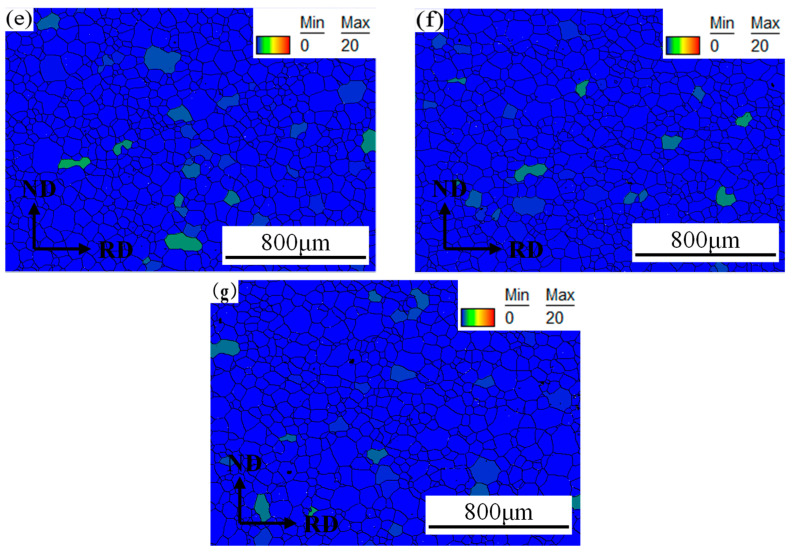
409L GOS spectra after annealing at different temperatures: (**a**) As-received; (**b**) 800 °C; (**c**) 840 °C; (**d**) 880 °C; (**e**) 910 °C; (**f**) 930 °C; (**g**) 950 °C.

Figure 3 shows the microstructure of 409L hot-rolled specimens after annealing at different temperatures. As shown in the figure, with the increase in annealing temperature, recrystallization nucleation and grain growth phenomena occur within the microstructure. When the annealing temperature is 800 °C, some nucleation sites are generated between the fiber bands, as shown in Figure 3a. When the annealing temperature is 840 °C, the ribbon-like fiber characteristics in the microstructure disappear, and most of the grains are equiaxed. As the annealing temperature further increases, the grains begin to grow, as shown in Figure 4b–f.

To clearly understand the recrystallization behavior, the degree of local orientation dispersion within the grain was investigated. Figure 4 shows the grain orientation spread (GOS) maps of 409L hot-rolled specimens after annealing at different temperatures, and the recrystallization fractions calculated based on GOS values are summarized in Table 1. It is generally considered that areas with GOS values < 1° are recrystallization areas [17]. As shown in the figure, with the increase in annealing temperature, the number of recrystallized grains with low GOS values gradually increases. When the annealing temperature is 800 °C, the recrystallization fraction increases from 2.1% in the hot-rolled state to 18.6%. When the annealing temperature reaches 840 °C, the recrystallization fraction sharply increases to 93.1%, leading to significant microstructural refinement of hot-rolled steel. As the annealing temperature further increases, recrystallization reaches a stable state, and some grain growth is also observed at higher annealing temperatures of 880–950 °C.

**Figure 5:**
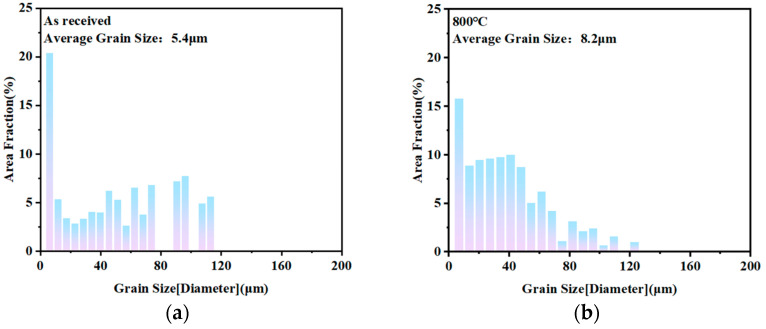

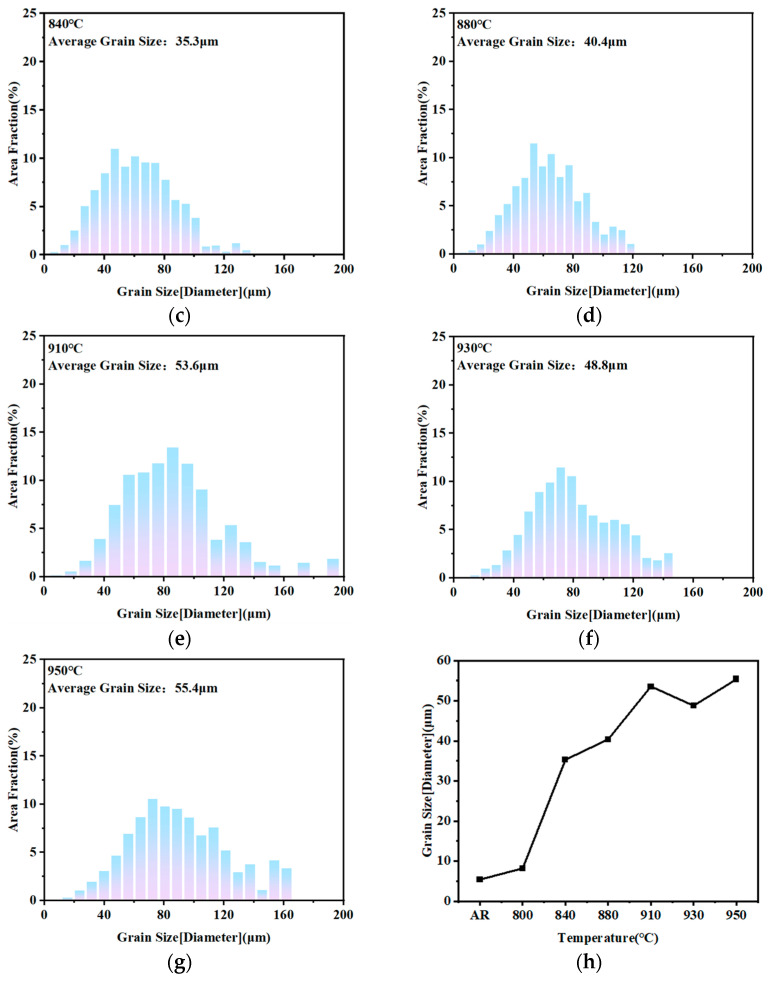
Grain size distribution of 409L hot-rolled specimens after annealing at different temperatures: (**a**) As-received; (**b**) 800 °C; (**c**) 840 °C; (**d**) 880 °C; (**e**) 910 °C; (**f**) 930 °C; (**g**) 950 °C; (**h**) average grain size at different annealing temperatures.

Figure 5 shows the grain size distribution of 409L hot-rolled specimens after annealing at different temperatures. To ensure the accuracy of grain size statistics, the grain size distribution under different annealing conditions is calculated by measuring the equivalent circle diameter of at least 500 grains in each specimen [12]. As shown in the figure, with the increase in annealing temperature, the average grain size of specimens gradually increases. This is because with the increase in temperature, the diffusion ability of atoms is enhanced, and the grain boundaries have a stronger migration ability. At this time, larger grains in the microstructure will take advantage of the relatively easy diffusion of atoms at the grain boundaries to continuously engulf surrounding small grains, thereby causing continuous self-growth [2]. When the annealing temperature exceeds 840 °C, some abnormally grown grains appear, resulting in an increase in average grain size and maintaining it near 55 μm after 910 °C.

### 3.2. Texture Evolution

**Figure 6:**
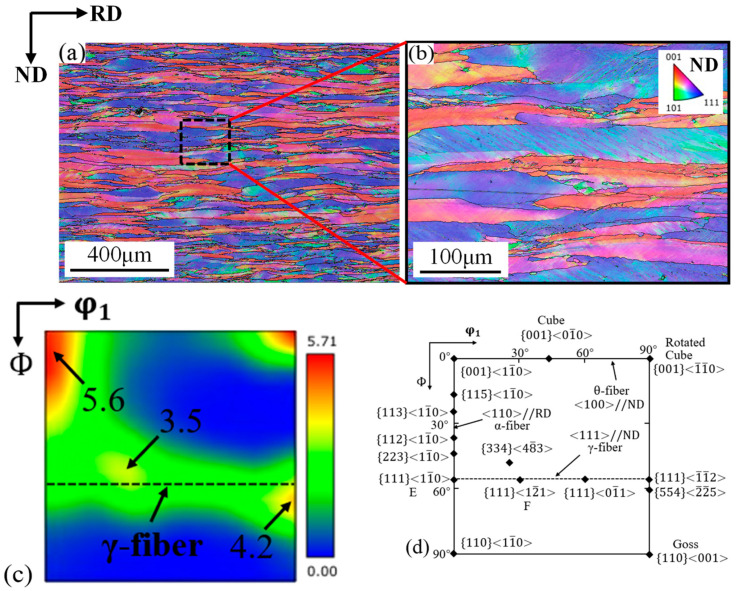
(**a**) ND-IPF of the FSS 409L hot-rolled sheet; (**b**) ND-IPF diagram at high magnification; (**c**) ODF of the FSS 409L hot-rolled specimen ($\varphi_{2}$ = 45°); (**d**) location of ideal orientations and fibers.

Figure 6a shows the ND-IPF of the center zone of 409L hot-rolled sheets. As seen in the figure, the texture of the hot-rolled specimen is mainly distributed in <001>//ND orientation and <111>//ND orientation, represented in red and blue colors, respectively. Also, as shown in Figure 6b, grains with shear bands can be clearly observed, and the main feature of these grains is γ-fiber texture, which is known as the preferred nucleation site for newly recrystallized grains [18]. Figure 6c shows the ODF of the center zone of the 409L hot-rolled sheet. As can be seen, the texture of 409L hot-rolled sheet consists of a strong α-fiber texture with {001}<110> component, and a relatively weak γ-fiber texture with {111}<112> component, with intensities of 5.6 and 4.2, respectively. For the main orientation {001}<110> in the α-fiber texture, due to its low energy storage and difficulty in recrystallization nucleation, the deformation texture is fully preserved after hot-rolling, resulting in a strong α-fiber texture [19]. On the other hand, the intensity of γ-fiber texture was developed mainly due to relatively low hot-rolling finishing temperature [17].

**Figure 7:**
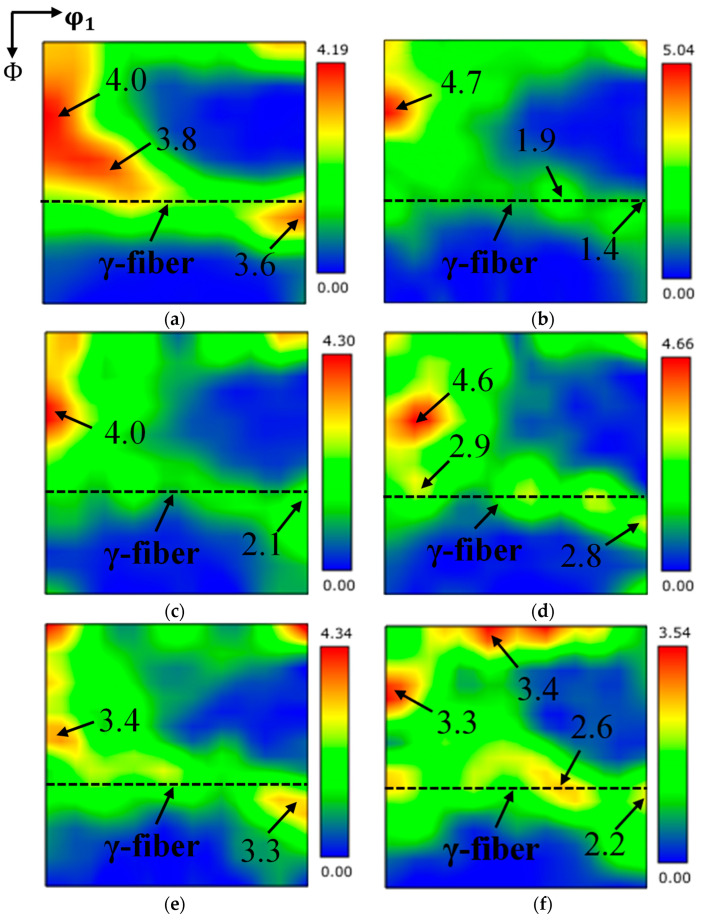
ODFs of the FSS 409L hot-rolled specimens ($\varphi_{2}$ = 45°) with different annealing temperatures: (**a**) 800 °C; (**b**) 840 °C; (**c**) 880 °C; (**d**) 910 °C; (**e**) 930 °C; (**f**) 950 °C.

Figure 7 shows the ODF of FSS 409L hot-rolled specimens with different annealing temperatures. The texture of hot-rolled specimens after annealing at the temperature range of 800–950 °C is mainly composed of a strong α-fiber texture and a relatively weak γ-fiber texture. The strongest α-fiber textures are mostly focused on {113}<110> and {001}<110>, and the strongest γ-fiber textures are concentrated in {111}<112> and {111}<001>. Compared with the hot-rolled texture, the α-fiber texture in the annealed specimens is significantly weakened, especially in the rotating cubic orientation {001}<110>. Due to the low Taylor factor, {001}<110> grain has a low recrystallization rate, and they will be separated and engulfed by the growth of other oriented grains around them during the annealing process [20]. Thus, a significant weakening of {001}<110> occurred during the annealing process. On the other hand, the intensity of the main {111}<112> component in γ-fiber texture is sharply decreased when annealed below 840 °C, and then increased slightly during annealing in the temperature range of 880–950 °C. During the annealing process, recrystallization nucleation preferentially occurs in areas with high energy storage (such as the shear band of the γ-fiber texture), forming new grains with different orientations [21]. When the annealing temperature is 800 °C, it is in the early stage of recrystallization (see in Figure 3 and Figure 4), and the grains with shear bands are replaced by new recrystallized grains, resulting in a decrease in the intensity of the main orientation {111}<112> in the γ-fiber texture compared to the untreated steels, from 5.6 to 3.6. At 840 °C, due to the near completion of recrystallization, the deformation zone was completely replaced by new recrystallized grains, leading to a further decrease in the intensity of the main orientation {111}<112> in the γ-fiber texture to 1.9. As the annealing temperature further increases, due to the high grain boundary energy of {111}<uvw> grains, the recrystallization nucleation rate and grain boundary migration rate increase [9], promoting significant development of γ-fiber textures with main orientations such as {111}<112>, {111}<011>, {111}<132>, and {554}<225>. When the annealing temperature is 950 °C, a strong Cube orientation {001}<010> appears, which to some extent reduces the intensity of the γ-fiber texture.

According to the mechanism of orientation nucleation and selective growth of recrystallization grains, the evolution of annealing texture is essentially an orientation competition driven by stored energy during recrystallization [22]. The orientation grains with high stored energy preferentially nucleate recrystallization grains. Specifically speaking, the order of stored energy of grain orientations is as follows: {110} > {111} > {112} > {100} component [23]. It is known that the {110}〈001〉 orientation will rotate along the following path:{110}〈001〉 → {001}〈110〉 → {554}〈225〉 → {111}〈112〉 → {111}〈110〉 [24]. As seen in Figure 7, a similar path can be observed in this study, where the α-fiber texture decreased with an increasing annealing temperature, while the original strong {554}〈225〉 texture became weakened, and {111}〈112〉 and {111}〈110〉 textures were formed. In contrast to {110}〈001〉, the metastable texture {111}<112> transforms into {111}<110> texture, which is more pronounced near complete recrystallization at 840–950 °C. This may be due to the migration of grain boundaries with high mobility, as discussed in the following section.

### 3.3. Grain Boundaries

**Figure 8:**
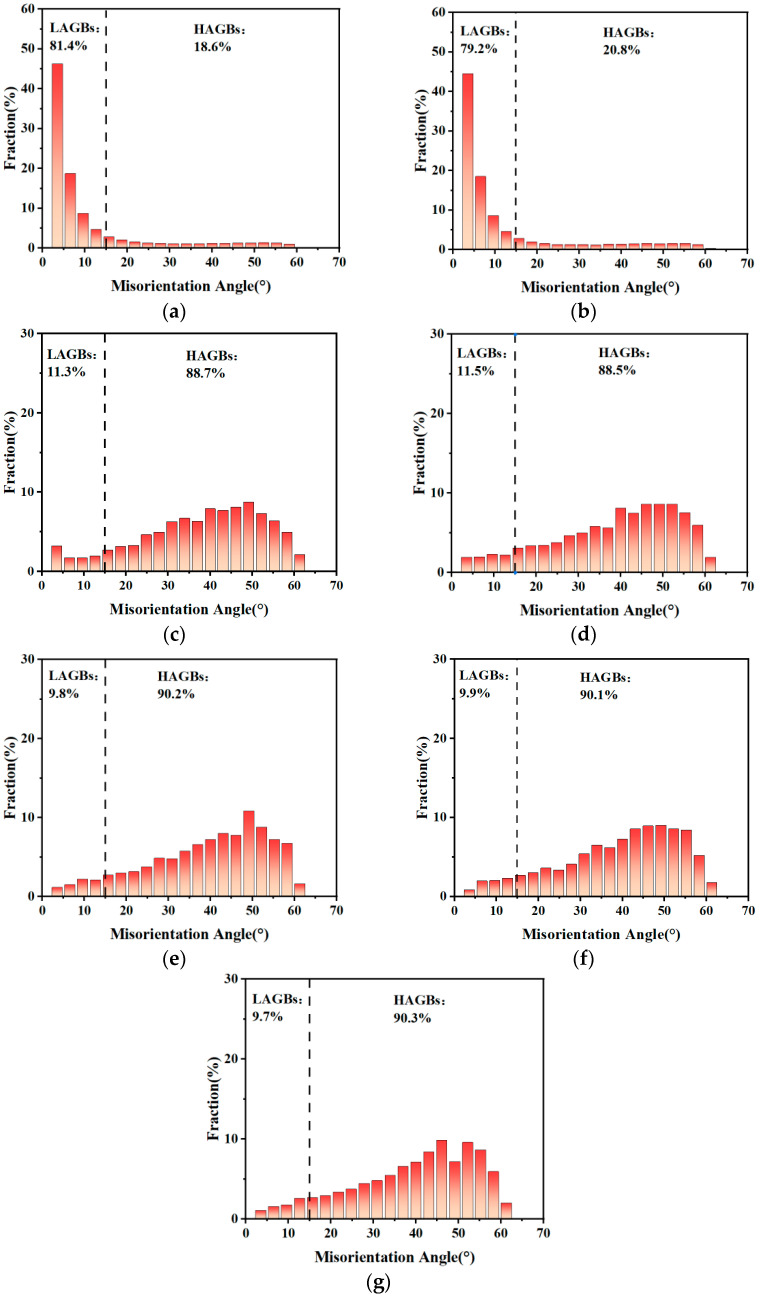
Misorientation distributions of 409L hot-rolled steels annealed at different temperatures: (**a**) As-received; (**b**) 800 °C; (**c**) 840 °C; (**d**) 880 °C; (**e**) 910 °C; (**f**) 930 °C; (**g**) 950 °C.

Figure 8 shows the misorientation distributions of 409L hot-rolled sheets annealed at different temperatures. For the as-received specimens, due to the formation of a large number of subgrains during the hot-rolling process [25], it is mainly characterized by low-angle grain boundaries (LAGBs, 2° < misorientation angles < 15°). The existing high-angle grain boundaries (HAGBs, misorientation angles > 15°) mainly originated from the accumulation of dislocations [26]. When annealed at 800 °C, which is the initial stage of recrystallization, the microstructure did not undergo significant changes, so the fraction of LAGBs and HAGBs did not change much. When the annealing temperature is increased to 840 °C, the vast majority of sub-grain boundaries disappear (see Figure 3b and Figure 4c), LAGBs can be evolved into HAGBs, and the growth of recrystallization grains is performed through the means of the migration of HAGBs [27]. When the annealing temperature is higher than 880 °C, the proportion of HAGBs further increases, and it reaches a maximum value of about 90% shown in Table 2, indicating that a uniform microstructure with a high recrystallization rate is formed in annealed specimens.

**Figure 9:**
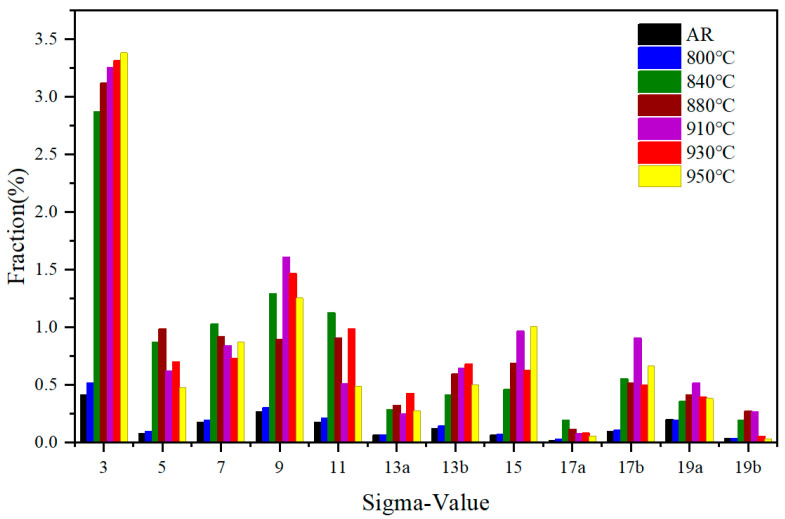
CSL boundary distribution of 409L hot-rolled steel annealed at different temperatures.

To investigate the growth of recrystallized grains during the annealing process of hot-rolled steel, the degree of the coincidence site lattice (CSL) boundaries was explored. The evolution of microstructure and texture strongly affects the misorientation of grain boundaries, leading to changes in CSL [28]. For the analysis of CSL boundaries, the boundary fraction was determined within the range of (∑3–∑49). For a clear view, only the CSL distribution within the range of (∑3–∑20) is displayed in the text shown in Figure 9, and the volume fraction of the prominent CSL boundaries for FFSs is summarized in Table 3. For the as-received steel, due to severe deformation during hot-rolling, it is difficult to maintain CSL grain boundaries, resulting in a very low proportion of 3.39%. As the annealing temperature increases, the total stored energy of the material increases, and the volume fraction of the CSL boundary sharply increases to 15.37% at 840 °C, and further increases and stabilizes at 16.5% after the temperature exceeds 880 °C. Specifically, the most prominent CSL boundary is ∑3, and its fraction increases significantly from the original 0.41 to 3.39% with increasing temperature. It is known that ∑3 is a low-energy and low-mobility boundary type defined by a rotation of 60°around a common axis <111>. This is the same rotation between the components with significant intensity in the ODF that belong to the same family planes and directions, such as (111) [1$\bar{1}$0]/(111) [0$\bar{1}$1] and (111) [$\bar{1}$21]/(1$\bar{1}$1) <112> [29]. During the hot-rolling process, the total energy of the material will increase due to the increase in dislocation density. In order to achieve the lowest energy configuration, the proportion of Σ3 grain boundaries significantly increases during recrystallization annealing [8,22]. For the high mobility grain boundaries, ∑5, ∑7, ∑9, ∑11, and ∑13b boundaries were analyzed [30]. As shown in Table 3, similar to the low mobility grain boundaries, the high mobility grain boundaries fraction of the original hot-rolled steel is very low compared to that at annealing. As the annealing temperature increases from 800 °C to 950 °C, the high mobility grain boundary fraction shows two upward and downward trends, with two different peaks, namely 4.74 at 840 °C and 4.56 at 930 °C. As is known, the degree of grain boundary coincidence affects the grain boundary energy, which in turn affects the grain boundary migration rate [31]. Based on the relationship between the driving energy of grain boundary migration and grain boundary energy ($P=\gamma_{b}/r$) [32], it can be known that the driving energy (*P*) required for grain boundary migration is directly proportional to the grain boundary energy ($\gamma_{b}$) and inversely proportional to the grain size (*r*). When the annealing temperature is 840 °C, the recrystallization ratio has significantly increased (see Figure 3 and Figure 4), providing sufficient driving energy for the simultaneous nucleation and growth of grains. Therefore, it can be considered that the effect of grain boundary energy is not significant at 840 °C, and the migration driving energy of grain boundaries mainly depends on grain size. It is obvious from Figure 5g that the average grain size at 840 °C is significantly lower than that at higher temperatures, resulting in a significant increase in the proportion of high mobility grain boundaries (i.e., 4.74%). When the annealing temperature exceeds 840 °C, the proportion of high mobility grain boundaries increases to 4.56 at 930 °C and sharply decreases to 3.6 at 950 °C, depending on the comprehensive effects of grain boundary energy ($\gamma_{b}$) and the grain size (*r*). In addition, it is noticed that the high mobility grain Σ13b boundary associated with the γ-fiber texture has a relatively low portion in the present study. During the annealing process from 800 °C to 930 °C, its proportion gradually increases from 0.15% to 0.68%, and then decreases to 0.5% at 950 °C.

### 3.4. Mechanical Properties and Formability

#### 3.4.1. Mechanical Properties

(1) Tensile properties

**Figure 10:**
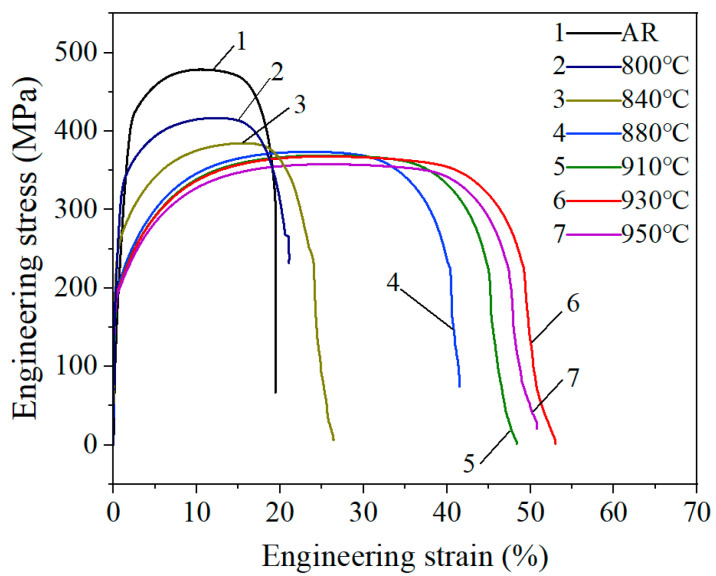
Stress–strain curve for the specimens at an angle of 0° to the rolling direction (RD).

**Figure 11:**
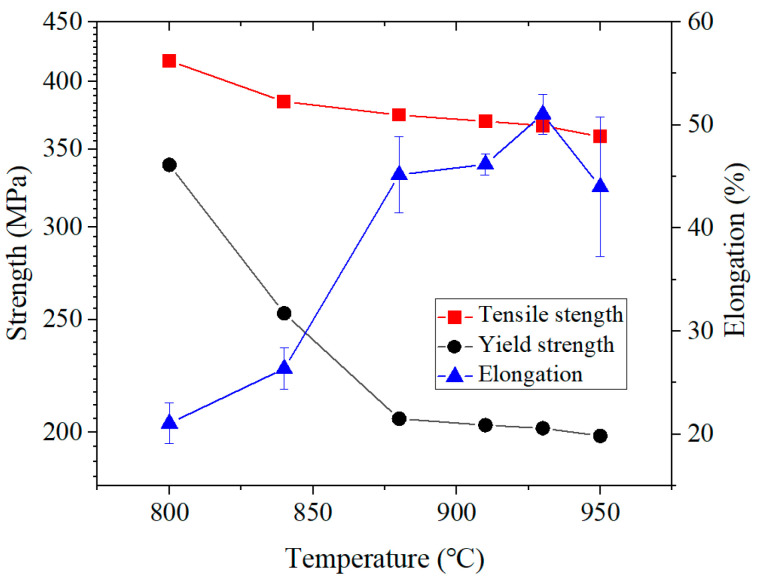
Strength and elongation of the 409L hot-rolled steel annealed at different temperatures.

The engineering stress–strain curves of 409L hot-rolled steels with different annealing temperatures are displayed in Figure 10, and their mechanical properties are presented in Table 4 and Figure 11. As seen in the curves, all specimens exhibited strain hardening and necking without a clear yield plateau. The stress value at 0.2% plastic strain was regarded as its yield strength. From the curve, it can be seen that the elongation of the as-received specimen and the annealed specimens in a relatively low temperature range (800–840 °C) is relatively low. When the annealing temperature is 880 °C, the elongation increases sharply, reaching a maximum of 51.5% at 930 °C. Then, as the temperature further increases to 950 °C, the elongation decreases to 44.1%. The tensile strength of the as-received specimen is 478.5 MPa and gradually decreases from 384.5 MPa to 358.7 MPa along with the annealing temperature from 800 to 950 °C. The yield strength exhibits a similar behavior to the tensile strength, gradually decreasing from the initial 425.4 MPa to 198.7 MPa annealed at 950 °C. The decreases in yield strength and tensile strength are mainly due to microstructural evolution during the annealing process, including nucleation and grain growth at high temperature. In Figure 10, it is evident that the area values under the engineering stress–strain curve of 930 °C, namely static toughness, are the highest, indicating that the specimen annealed at 930 °C has the best comprehensive mechanical properties. This optimal match of these comprehensive mechanical properties may be caused by the development of important texture components [33] discussed in the following Section 3.4.2.

(2) Fracture morphology

**Figure 12:**
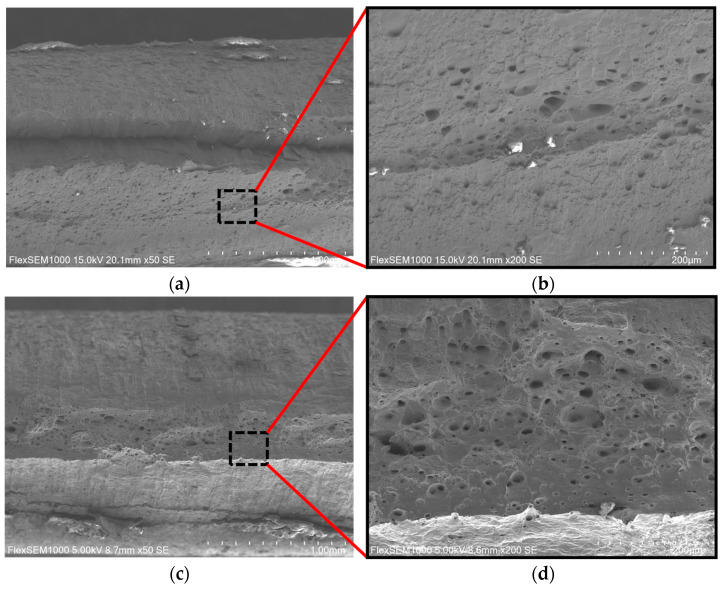

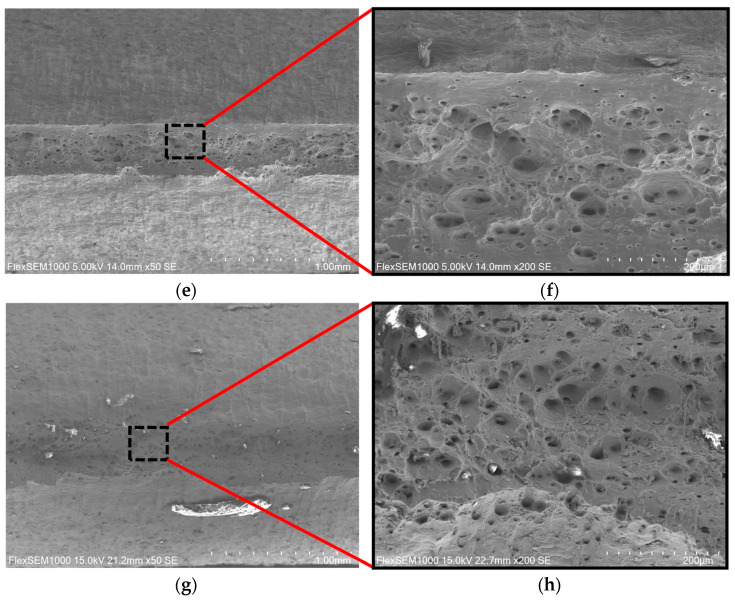
Fracture characteristics of 409L hot-rolled steels annealed at different temperatures: (**a**) As-received; (**b**) as-received magnified view; (**c**) 800 °C; (**d**) 800 °C magnified view; (**e**) 840 °C; (**f**) 840 °C magnified view; (**g**) 910 °C; (**h**) 910 °C magnified view.

Figure 12 shows SEM images of the tensile fracture surfaces of the 409L hot-rolled tensile specimen after different annealing temperatures. As shown in Figure 12a,b, the fracture surface of the hot-rolled tensile specimen is relatively flat, with some small and shallow dimples. It exhibits a mixed fracture characteristic of quasi-cleavage fracture and ductile fracture, indicating poor plasticity of the material, as evidenced by its low elongation [34].

As shown in Figure 12c–f, after annealing, the fracture surfaces of the tensile specimens all exhibited obvious characteristics of ductile fracture. The microscopic morphology of the fracture surface is mainly characterized by equiaxed dimples of various sizes, which can effectively reduce stress concentration at the crack and inhibit crack propagation [35]. As the annealing temperature increases, it can be clearly observed that the size of the dimples has increased and their number has grown. Compared to the condition at 840 °C, after annealing at 910 °C, the dimple distribution is more uniform, indicating that the material has been subjected to more extensive plastic deformation, and the plasticity continues to improve, consistent with the elongation trend in Figure 11.

#### 3.4.2. Formability

(1) Strain hardening exponent

For ferritic stainless steel, the strain hardening exponent (*n*) is a critical factor in controlling formability. A higher *n*-value indicates better formability, reducing the risk of thinning or cracking during deformation [36,37]. Lower *n*-values (e.g., 0.1–0.2) typically lead to faster strain hardening rates, which can cause localized thinning or necking during deformation [38]. To determine the strain hardening exponent, the Hollomon equation describing the relationship between true stress and true strain was used, as shown below:

(1) $$\sigma =k\varepsilon^{n}$$

where *n* is the strain hardening exponent and k is the strength coefficient. After taking logarithms of both sides of Equation (1), the *n*-value can be expressed as follows:

(2) $$n=\frac{d(ln\sigma )}{d(ln\varepsilon )}$$

**Figure 13:**
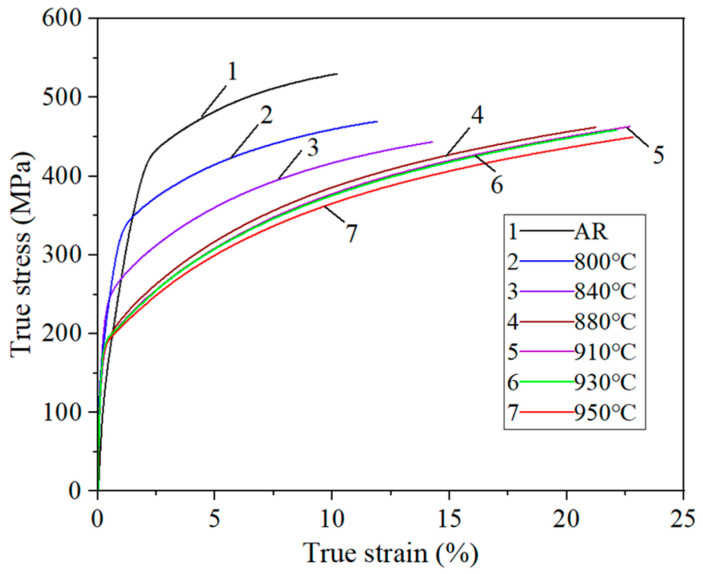
True stress–strain curve (range before ultimate strength point) for the specimens at an angle of 0° to the rolling direction (RD).

**Figure 14:**
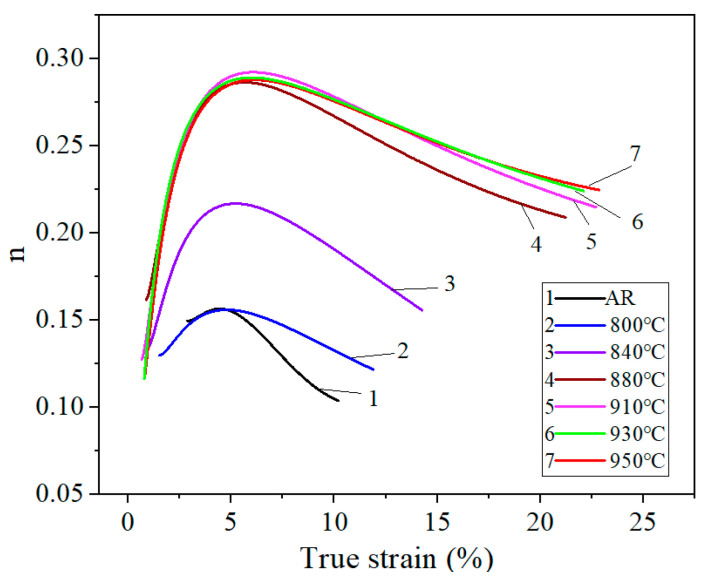
The variation in *n*-value at the uniform plasticity stage of the true stress–strain curve.

Figure 13 shows true strain–stress curves before the ultimate strength. By fitting lnσ as a function of lnε using a fifth-order equation, *n*-values in the uniform plasticity stage of the true stress–strain curve (i.e., range from yield point to ultimate strength) can be calculated, and the *n*-values as a function of true strain are presented in Figure 14. As seen in the figure, within the entire uniform plasticity range, the value of *n* shows a trend of first increasing and then decreasing in all specimens, and it rises as annealing temperature increases. For the as-received and the specimen annealed at 800–840 °C, the *n*-value is in a relatively small range (less than 0.2), leading to poor formability by the high strain hardening rate induced from severe deformation during hot-rolling [35]. However, it was observed that above 880 °C, the *n*-value rises sharply, peaking at over 0.28, indicating superior formability. In addition, the true strain value at which uniform plastic deformation begins decreases with temperature, as can be seen from the curves shifting to the left. Generally, the *n*-value is related to the dislocation density and grain size in the material. The annealing treatments can reduce pile-up of dislocations and refine grain boundaries by recrystallization, leading to a higher *n*-value and better formability. In this study, the strain hardening behavior at 930 °C and 950 °C was very similar, with the highest *n*-value, exhibiting the best formability.

(2) Plastic strain ratio

Other key indicators for evaluating the formability of FSSs are the average plastic strain ratio ($\bar{r}$) and the anisotropy index (∆r). The average plastic strain ratio and the anisotropy index after annealing at different temperatures can be calculated by the following equation:

(3) $$\bar{r}=\frac{r_{0}+r_{90}+2r_{45}}{4}$$

(4) $$∆r=\frac{r_{0}+r_{90}-2r_{45}}{2}$$

where $r_{0}$, $r_{45}$, and $r_{90}$ represent the plastic strain ratios of specimens at 0°, 45°, and 90° angles, respectively.

**Figure 15:**
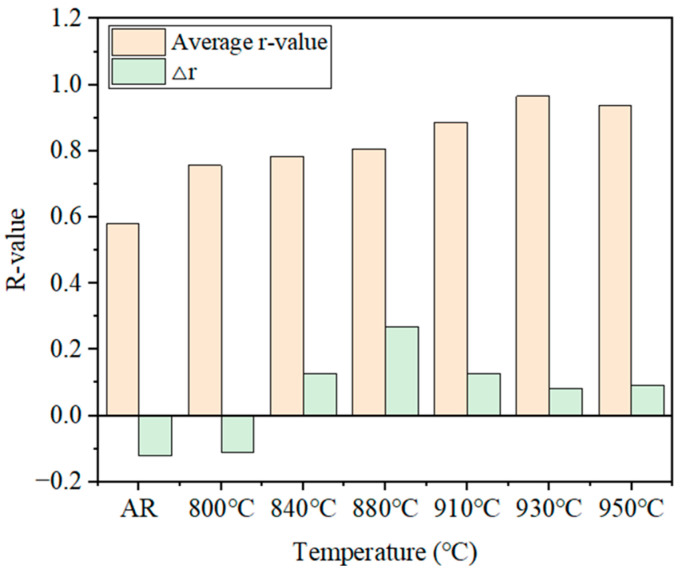
Average plastic strain ratio ($\bar{r}$) of the 409L hot-rolled steels annealed at different temperatures.

Table 5 and Figure 15 summarize detailed *r*-values of hot-rolled steels under different annealing conditions. The results indicated that *r*-values in all directions generally show an increasing tendency with increasing temperature. The average plastic strain ratio $\bar{r}$ of the hot-rolled specimen is 0.58, and it gradually increases and then decreases, as shown in Figure 15. The maximum average *r*-value of 0.96 was obtained at 930 °C, which was 67% higher than that of the untreated specimen. At the same time, the anisotropy index ∆r is relatively low at this temperature, indicating optimal formability of 409L hot-rolled steels under this annealing condition. As the annealing temperature further increased, the average *r*-value decreased to 0.93.

The formability of ferritic stainless steel is correlated with recrystallization texture and microstructure [39]. A uniform and strong γ-fiber recrystallization texture, along with a uniform grain size distribution, is known to improve the formability of FSSs [26]. From Figure 4 and Figure 7, it can be seen that during the recrystallization transition stage at relatively low annealing temperatures (800~840 °C), the specimen exhibits uneven microstructure and size distribution; accordingly, the formability of the specimens at this temperature range shows relatively low $\bar{r}$-values. When the annealing temperature exceeds 840 °C, the uniform and strong recrystallization textures are observed along with an even distribution of grain sizes (Figure 4c–g). In this stage, the formability of materials is mainly governed by recrystallization texture.

**Figure 16:**
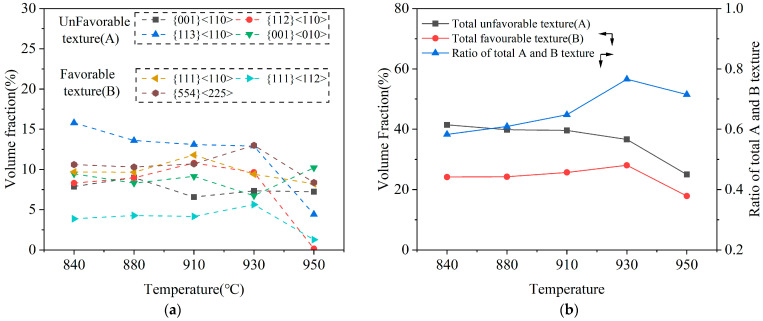
(**a**) The volume fraction of the main textures in 409L hot-rolled sheet annealed at different temperatures; (**b**) volume fraction of total unfavorable and favorable textures.

**Figure 17:**
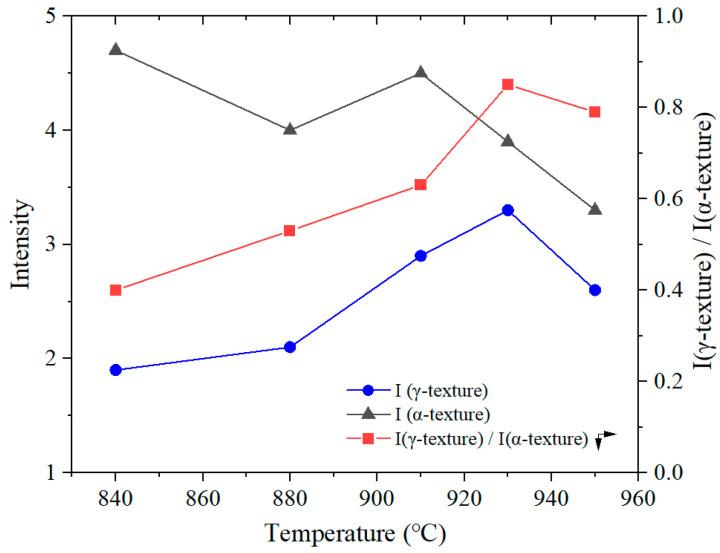
The variation in intensity of the *α*-texture and *γ*-texture ({111}) in 409L hot-rolled sheet annealed at different temperatures.

**Figure 18:**
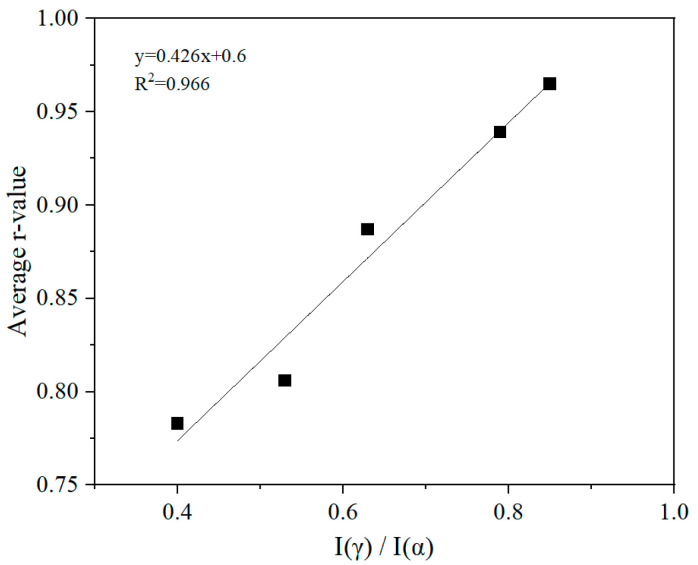
Correlation between average $\bar{r}$-value and $I_{\left(\gamma \right)}/I_{\left(\alpha \right)}$ texture ratio in 409L hot-rolled sheet after annealing at different temperatures.

To better understand the influence of texture on the formability, a quantitative analysis was conducted on the evolution of the main texture during the annealing process. Figure 16 shows the volume fraction of the main textures in the specimens annealed at different temperatures [40,41]. To exclude the effect of dislocation density on texture evolution, only the texture within the range of 840–950 °C was considered in the analysis. When the annealing temperature increases, the total proportion of unfavorable texture (represented as A in Figure 16), mainly including {001}<110>, {112}<110>, {113}<110>, and Cube {001}<010> components, decreased significantly from 41.4% at 840 °C to 25.0% at 950 °C. On the other hand, the total proportion of favorable textures, including {111}<110>, {111}<112>, and {554}<225> components, gradually increased, reaching a maximum of 28.1% at 930 °C by forming relatively strong {554}<225> textures, and then decreased to 17.9% at 950 °C. Figure 17 shows the variation in intensity of the α-texture and γ-texture ({111}) in the 409L hot-rolled sheet with annealing temperatures. As seen in the figure, the intensity of α-texture shows a decreasing tendency within the overall temperature range, while that of γ-texture gradually increases first and reaches its maximum at 930 °C and then sharply decreases at 950 °C. It was found that the intensity of γ-texture at 950 °C is lower than that at 910 °C, which is definitely different from the change in the average plastic strain ratio shown in Figure 15. However, the variation behavior of I(γ)/I(α) in the specimens shown in Figure 17 is very similar to that of the average plastic strain ratio. Also, as shown in Figure 18, the I(γ)/I(α) texture ratio is positively correlated with the average plastic strain ratio, with a linear fitting coefficient ($R^{2}$) greater than 0.95. Therefore, it can be concluded that the formability of tested steels mainly depends on the relative intensity of the γ-texture and α-texture, rather than just γ-texture intensity.

## 4. Conclusions

The microstructure, texture, and mechanical properties, as well as plastic strain ratio (r-value) of the 409L hot-rolled ferritic stainless steel with a strong α-fiber texture were investigated at different annealing temperatures. Recrystallization was mostly completed in the grains of steels annealed at 880–950 °C. The annealing process of hot-rolled steels was observed to substantially increase the ∑3 boundaries, while showing no significant effect on the ∑13b boundaries. As the annealing temperature increases, tensile strength slowly decreases, while the elongation first increases and then decreases. The maximum static toughness of the specimen is obtained at 930 °C. The intensity of α-fiber texture generally decreases with increasing temperature, while the intensity of γ-fiber texture decreases at the initial stage of recrystallization below 840 °C and then increases at 880–950 °C. The hot-rolled steels annealed at 930 °C have the highest $\bar{r}$ and lowest ∆r values, mainly attributed to the uniformity of microstructure and the maximum I(γ)/I (α) texture strength ratio, thus improving formability and facilitating subsequent cold-rolling.

## Figures and Tables

**Table 1:** Recrystallization fraction of 409L hot-rolled specimens with different annealing temperatures.

Temperature/°C	As-Received	800	840	880	910	930	950
Recrystallized fraction/%	2.1	18.6	93.1	96.1	95.3	97.5	96.5

**Table 2:** Volume fractions of LAGBs and HAGBs of 409L hot-rolled steels at different annealing temperatures.

Temperature/°C	As-Received	800	840	880	910	930	950
Volume fraction of LAGBs/%	81.4	79.2	11.3	11.5	9.8	9.9	9.7
Volume fraction of HAGBs/%	18.6	20.8	88.7	88.5	90.2	90.1	90.3

**Table 3:** Volume fraction of CSL boundaries of 409L hot-rolled sheets at different annealing temperatures.

Temperature/°C	AR	800	840	880	910	930	950
Volume fraction of CSL boundaries/%	3.39	3.77	15.37	16.09	16.52	16.47	16.50
Low mobility (∑3) boundaries/%	0.41	0.52	2.87	3.12	3.26	3.31	3.39
Low mobility (∑9) boundaries/%	0.27	0.30	1.29	0.90	1.61	1.47	1.26
Low mobility (∑11) boundaries/%	0.17	0.21	1.13	0.90	0.51	0.98	0.49
Low mobility (∑13b) boundaries/%	012	0.15	0.41	0.60	0.65	0.68	0.50
High mobility (∑5\∑7\∑9\∑11\∑13b) Boundaries/%	0.82	0.95	4.74	4.3	4.22	4.56	3.60

**Table 4:** Mechanical properties of hot-rolled steels under different annealing conditions.

Annealing Process	Yield Strength (MPa)	Tensile Strength (MPa)	Elongation (%)
AR	425.4 ± 0.7	478.5 ± 0.7	19.5 ± 2.5
800 °C for 3 min.	339.4 ± 0.8	416.7 ± 0.8	21.1 ± 1.7
840 °C for 3 min.	253.1 ± 0.7	384.5 ± 0.7	26.4 ± 2.1
880 °C for 3 min.	205.5 ± 0.8	374.5 ± 0.8	45.2 ± 3.7
910 °C for 3 min.	202.9 ± 0.9	369.9 ± 0.9	46.2 ± 1.0
930 °C for 3 min.	201.7 ± 1.1	366.7 ± 1.1	51.1 ± 2.0
950 °C for 3 min.	198.7 ± 0.9	358.7 ± 0.9	44.0 ± 6.7

**Table 5:** $\bar{r}$ and ∆r values of hot-rolled steels under different annealing conditions.

Annealing Process	$r_{0}$	$r_{45}$	$r_{90}$	$\bar{r}$	∆r
AR	0.39	0.64	0.65	0.58	−0.12
800 °C for 3 min	0.61	0.81	0.79	0.76	−0.11
840 °C for 3 min	0.75	0.72	0.95	0.78	0.13
880 °C for 3 min	0.79	0.67	1.09	0.81	0.27
910 °C for 3 min	0.84	0.82	1.06	0.89	0.13
930 °C for 3 min	0.7	0.92	1.31	0.97	0.08
950 °C for 3 min	0.73	0.89	1.23	0.94	0.09

## Data Availability

The original contributions presented in this study are included in the article. Further inquiries can be directed to the corresponding authors.
